# Reduced peak stimulated growth hormone is associated with hyperuricemia in obese children and adolescents

**DOI:** 10.1038/s41598-018-26276-w

**Published:** 2018-05-21

**Authors:** Shuang Liang, Dejian Zhang, Jianhong Qi, Xiaobo Song, Jiang Xue

**Affiliations:** 1 Department of Pediatrics, The Second Hospital of Shandong University, Shandong, China; 2 Department of Pediatrics, Shandong Provincial Hospital Affiliated to Shandong University, Shandong, China; 3 Department of Pediatrics, Children’s Hospital of Changchun, Jilin, China

## Abstract

The purpose of the study is to investigate whether reduced peak GH response to arginine-levodopa test is associated with hyperuricemia in obese children and adolescents. The study population consisted of a total sample of 78 obese and 30 normal-weight children and adolescents without known hypopituitarism. All participants underwent clinical examination and GH stimulation testing. IGF-1, lipid profile and other metabolic markers were assessed. The obese subjects were then divided into two groups according to the serum levels of uric acid. Results show that obese subjects had significantly lower peak GH, lower IGF-1 and similar height SDS than those in the control group. Children with hyperuricemia had significantly lower peak stimulated GH compared with non-hyperuricemia obese subjects. Results from logistic regression model showed that peak GH were negatively associated with hyperuricemia after controlling for age, gender, tanner stage, BMI SDS, IGF-1, blood pressure, HOMA-IR, lipids status. These data indicate that lower peak GH is positively associated with hyperuricemia in obese children and adolescents without known hypothalamic/pituitary disease after controlling BMI and insulin resistance, as well as other cardiometabolic risk factors.

## Introduction

As one of the recognized public health problems, childhood obesity has reached epidemic levels globally. Apart from multiple obesity-related metabolic complications, it has been firmly established that obesity is characterized by the reduced basal and pulsatile release of growth hormone(GH)^1–3^ as well as the decreased stimulated GH release^4–6^ in both children and adults. This GH deficiency associated with obesity is relative and is reversible with weight loss^3,7^. During the last decades, several studies indicated that this functional hyposomatotropism was associated with cardiometabolic risk markers in obesity. For example, Makimura *et al*.^8^ demonstrated a significant negative association between GH secretion and carotid intima-media thickness (cIMT) in obese men and women. Similarly, Utz *et al*.^9^ also found that relative GH deficiency of obesity was associated with cIMT, increased cardiovascular risk and insulin resistance based on a sample of 45 overweight and obese women. Likewise, by investigating the situation of premenopausal women with obesity, peak GH was found to be inversely associated with intramyocellular and intrahepatic lipid content^10^. Finally, similar studies proved the existence of independent effects of reduced GH secretion on cardiovascular disease risk among obese without structural pituitary disease^6,11,12^.

Uric acid is the major end product of purine metabolism and is excreted in the urine. In previous studies, elevated serum uric acid levels have been associated with obesity, insulin resistance and metabolic syndrome^13^. Furthermore, as an independent risk factor for cardiovascular disease, serum uric acid is associated with cIMT in asymptomatic prepubertal children with higher body mass index (BMI) or more preperitoneal fat^14^. In a recent study, it was confirmed that hyperuricemia can be an early marker of cardiovascular dysfunction and the routine determination in pediatric obesity^15^. Despite many evidences admitting that uric acid is a cardiometabolic risk factor, and obesity-related hyposomatotropism is associated with classical risk factors for cardiovascular disease, to best of our knowledge, studies focusing on the association between obesity-related hyposomatotropism and hyperuricemia are still scanty. Accordingly, the purpose of this study is to investigate the association between peak stimulated GH and serum uric acid level in obese children and adolescents.

## Results

### Clinical characteristics of study subjects

Clinical characteristics for the 78 obese children, and 30 healthy controls are shown in Table 1. Obese subjects had lower peak GH on the arginine-levodopa stimulation test, and it also had lower insulin-like growth factor 1 (IGF-1) and high density lipoprotein cholesterol (HDL-C) compared with the control group. Subjects in the obese group had higher uric acid, higher levels of serum insulin, total cholesterol (TC), low density lipoprotein cholesterol (LDL-C), triglycerides (TG) and alanine aminotransferase (ALT) compared with normal-weight controls. BMI standard deviation scores (SDS), systolic blood pressure (SBP), diastolic blood pressure (DBP), homeostasis model assessment of insulin resistance (HOMA-IR) were also significantly higher in the obese group than those in the control group. There were no significant differences in terms of age, gender, tanner stage, height SDS, fasting glucose, urea nitrogen and creatinine.

**Table 1:** Clinical and laboratory characteristics of study participants. *P < 0.05.

Characteristics	Obese group (n = 78)	Control group (n = 30)	P value
Age (yr)	12.24 (1.38)	11.7 (1.95)	0.17
Male/Female (n)	69/9	22/8	0.08
Prepuberty/Puberty, (n)	57/21	16/14	0.07
BMI SDS	2.51 (0.9)	−0.11 (0.87)	<0.001*
Height SDS	0.18 (1.15)	0.14 (0.8)	0.84
SBP (mmHg)	117.44 (14.3)	105.6 (8.84)	<0.001*
DBP (mmHg)	78.44 (11.43)	69.43 (7.3)	<0.001*
Peak GH (μg/L)	4.08 (3.88)	16.31 (4.43)	<0.001*
IGF-1	268.36 (146.05)	390.25 (168.33)	<0.001*
uric acid (mg/dL)	6.42 (1.87)	5.01 (1.3)	<0.001*
HOMA-IR	5.9 (4.68)	2.19 (1.18)	<0.001*
Insulin (uU/mL)	25.83 (19.8)	9.84 (5.34)	<0.001*
Fasting glucose (mmol/L)	5.08 (0.48)	5 (0.35)	0.45
TC (mmol/L)	4.69 (1.15)	3.92 (0.6)	<0.001*
HDL-C (mmol/L)	1.29 (0.33)	1.47 (0.29)	0.005*
LDL-C (mmol/L)	2.72 (0.9)	2.16 (0.45)	<0.001*
TG (mmol/L)	1.51 (1.11)	0.79 (0.38)	<0.001*
ALT (U/L)	39.1 (47.69)	14.73 (5.45)	<0.001*
urea nitrogen (mmol/L)	4.34 (0.82)	4.5 (1)	0.56
creatinine (μmol/L)	46.59 (10.71)	45.84 (10.71)	0.83

Among 78 enrolled obese subjects, 69 (88.5%) children were male. Average value of BMI SDS for the cohort was 2.51 ± 0.9. Hyperuricemia was identified in 45 subjects (57.7%). Relative GHD was observed in 70 subjects (89.7%). Metabolic syndrome was identified in 22 subjects (28.2%). The prevalence rates (PR%) of metabolic syndrome components, including hypertriglyceridemia, low HDL-C, elevate blood pressure and elevated FPG, were 26.9%, 15.4%, 37.2%, and 14.1% respectively.

### Peak stimulated GH and hyperuricemia

Clinical characteristics and laboratory findings of the obese subjects stratified according to serum levels of uric acid, which are shown in Table 2. Obese subjects were separated into two subgroups according to serum levels of uric acid. Children with hyperuricemia had lower peak stimulated GH and HDL-C, and higher values for HOMA-IR, insulin, TG, ALT than subjects with no hyperuricemia. Age, gender, tanner stage, Pituitary height, BMI SDS, height SDS are similar between the two patient groups. The levels of serum IGF-1, TC, LDL-C, fasting glucose, urea nitrogen and creatinine were also similar in these two groups.

**Table 2:** Clinical and laboratory characteristics in the two groups according to serum levels of uric acid. *P < 0.05.

Characteristics	Hyperuricemia (n = 45)	Non-hyperuricemia (n = 33)	P value
Age (yr)	12.38 (1.37)	12.05 (1.38)	0.3
Male/Female (n)	42/3	27/6	0.16
Prepuberty/Puberty, (n)	32/13	25/8	0.80
Pituitary height	4.85 (1.22)	4.63 (1.30)	0.46
BMI SDS	2.65 (0.85)	2.32 (0.96)	0.19
Height SDS	0.2 (1.09)	0.15 (1.24)	0.83
SBP (mmHg)	117.82 (14.82)	116.91 (13.76)	0.78
DBP (mmHg)	77.93 (10.63)	79.12 (12.57)	0.65
Peak GH (μg/L)	2.91 (2.73)	5.68 (4.63)	0.001*
IGF-1	258.15 (134.69)	282.29 (161.36)	0.47
HOMA-IR	6.84 (5.57)	4.62 (2.65)	0.03*
Insulin (uU/mL)	29.63 (23.61)	20.64 (11.34)	0.04*
Fasting glucose (mmol/L)	5.11 (0.45)	5.04 (0.52)	0.51
TC (mmol/L)	4.8 (1.38)	4.52 (0.71)	0.42
HDL-C (mmol/L)	1.23 (0.36)	1.37 (0.27)	0.02*
LDL-C (mmol/L)	2.84 (1.07)	2.57 (0.58)	0.29
TG (mmol/L)	1.78 (1.36)	1.14 (0.46)	0.007*
ALT (U/L)	49.24 (59.34)	25.27 (17.04)	0.02*
urea nitrogen (mmol/L)	4.35 (0.89)	4.71 (1.11)	0.11
creatinine (μmol/L)	47.83 (11.50)	44.9 (9.43)	0.31

Logistic regression analysis was performed to assess the relationship between peak stimulated GH and hyperuricemia. Notably, peak stimulated GH was significantly associated with hyperuricemia after controlling for age, gender, tanner stage, BMI SDS, IGF-1, blood pressure, HOMA-IR, lipids status for overall model (OR0.689, 95% CI 0.503–0.944; P = 0.02).

### Relationship between peak stimulated GH and cardiometabolic variables

Peak GH after stimulation with arginine and levodopa was significantly inversely associated with uric acid (r = −0.39, P <0.001). In addition, peak stimulated GH was negatively associated with traditional cardiovascular disease risk factors such as BMI SDS(r = −0.41, P < 0.001), SBP(r = −0.289, P = 0.01), DBP(r = −0.317, P = 0.005), HOMA-IR(r = −0.344, P = 0.002), insulin(r = −0.359, P = 0.001), TC(r = −0.242, P = 0.03), LDL-C(r = −0.322, P = 0.004), as well as TG(r = −0.232, P = 0.04). Peak GH is also positively associated with HDL-C(r = 0.341, P = 0.02) (see Table 3), while no association was found between peak stimulated GH and fasting glucose.

**Table 3:** Correlation between GH peak and parameters of cardiovascular risk marker. *P < 0.05.

Variable	r	p
BMI SDS	−0.41	<0.001*
SBP (mmHg)	−0.289	0.01*
DBP (mmHg)	−0.317	0.005*
uric acid(mg/dL)	−0.39	<0.001*
HOMA-IR	−0.344	0.002*
Insulin(uU/mL)	−0.359	0.001*
TC (mmol/L)	−0.242	0.03*
HDL-C (mmol/L)	0.341	0.002*
LDL-C (mmol/L)	−0.322	0.004*
TG (mmol/L)	−0.232	0.04*
Fasting glucose (mmol/L)	−0.03	0.8

### Peak stimulated GH and the metabolic syndrome

We applied logistic regression models adjusted for age, gender and tanner stage to determine the association between the peak stimulated GH and the metabolic syndrome. Results show that peak stimulated GH was associated with a decreased possibility of having the metabolic syndrome (OR0.755, 95% CI 0.577–0.988; P = 0.04).

## Discussion

In this study, we demonstrated that obese children had reduced peak stimulated GH, lower IGF-1 and normal stature. Furthermore, we provided evidences that lower peak GH is positively associated with hyperuricemia in obese children and adolescents without known hypothalamic/pituitary diseases after controlling BMI and insulin resistance, as well as other cardiometabolic risk factors.

In our study, we observed a significant association between peak stimulated GH and hyperuricemia in Chinese obese children and adolescents. First, hyperuricemia patients had reduced peak stimulated GH. Second, peak stimulated GH was significantly correlated with serum uric acid level. Third, more importantly, an independent correlation between peak stimulated GH and hyperuricemia was confirmed on multivariate regression analysis by controlling several confounders including anthropometric, hormonal factors and cardiometabolic variables. To the best of our knowledge, this study should be one of the first attempts to investigate the relationship between peak GH and hyperuricemia.

Although the mechanisms for the relationship between decreased peak stimulated GH and hyperuricemia has not been elucidated, it may be explained by the following reasons. First, uric acid is significantly associated with the serum leptin concentration^16,17^. Some studies showed that leptin could be a pathogenic factor responsible for hyperuricemia in obese patients^13^. On the other hand, Kirsz *et al*.^18^ found that leptin have direct suppressed effects on somatotropin secretion in animal model. In another study in adults, Li *et al*.^19^ observed that leptin were independent risk factors for GHD. As hyperuricemia is closely related to the serum leptin level, the negative correlation between leptin and GH may indirectly support the relationship between low peak GH and hyperuricemia. Second, another possible explanations for this relationship is that insulin resistance is the link between GH and uric acid. Hyperuricemia is suggested to be associated with insulin resistance^20^. Recent studies found that high uric acid directly inhibited insulin signal and induces insulin resistance^21^. Insulin resistance can not only increase uric acid synthesis^22^, but also decrease uric acid excretion^23^. Low GH level is also associated with insulin resistance. Hyperinsulinemia have a direct inhibitory effect on GH secretion^24,25^, and more severe insulin resistance may impair Growth Hormone Releasing Hormone (GHRH) and the arginine-induced GH response in a population of adults aged 50–90^11^. The third mechanism may be related to the fact that uric acid is significantly and independently associated with oxidative and inflammatory alterations^25,26^. Reduced growth hormone secretion was also associated with increased chronic inflammation in obese subjects without known pituitary diseases^8,9^, and lower GH levels often accompany with oxidative stress^27^. Moreover, Fukushima *et al*.^28^ found that growth hormone administration could decrease oxidative stress, and chronic inflammation thus ameliorates glucose intolerance in obese mice. Accumulated evidence has shown that oxidative and inflammatory maybe the link between the reduced peak stimulated GH and hyperuricemia.

Hyperuricemia and obesity-related relative GH deficiency (GHD) share many similar features. Hyperuricemia has been shown to be associated with an increased risk of cardiovascular disease^29^, we have also confirmed in our study that children with hyperuricemia had higher values for HOMA-IR, insulin, TG and lower HDL-C compared with subjects with no hyperuricemia. Previous studies have established that low endogenous GH secretion was associated with increased cardiovascular risk markers in obesity^4,6,8–12^. In present study we also confirmed the association of peak stimulated GH on BMI SDS, blood pressure, HOMA-IR, TC, TG, HDL-C, LDL-C as well as the metabolic syndrome. These results are in line with previous findings and extend these results to obese children and adolescents. Based on this point, strategies may be also beneficial to GH administration in patients with hyperuricemia.

Our study has potential limitations that must be considered. First, we cannot complete exclude the possibility that patients with true GHD had higher BMI. To reduce this possibility, we excluded children with short stature and performed hypothalamic pituitary Magnetic Resonance Imaging (MRI) in all obese children. Second, as a cross-sectional study, the definitive cause and effect relationship cannot be inferred. Third, the study group consisted of only Chinese children, limiting the generalization of these conclusions. Finally, in this study, we do not have data on serum inflammatory markers and waist circumference.

This study also has several strengths. First, to the best of our knowledge, our investigation is the one of the first attempts to document an inverse relationship between peak stimulated GH levels and serum uric acid concentration independent of several confounding factors. Second, all subjects received similar GH provocative tests in order to eliminate the wide variability in peak GH response. Third, our study had a relatively large sample size of children with detailed clinical characterization. Finally, several confounding variables had been reported to affect GH levels. In the current study, in order to limit the confounding effects, we matched children for age, gender and tanner stage in study population.

In conclusion, for children with obesity, GH response to arginine-levodopa testing is reduced in obese subjects. And our study confirmed a strong inverse relationship between peak GH and hyperuricemia independent of several confounders. In addition, data from this study demonstrated that peak stimulated GH is associated with cardiovascular risk factors, including obesity, insulin resistance, low levels of HDL-cholesterol, elevated triglycerides, hypertension and metabolic syndrome. Reduced peak stimulated growth hormone may play an independent role in hyperuricemia, increasing cardiovascular disease risk markers and metabolic syndrome in obesity. Thus, our findings suggested that additional investigation for GH status might be warranted in obese subjects and was considered in the clinical evaluation of their metabolic risk profile. It is not fully understood whether GH replacement in this subpopulation of obese patients is useful to improve hyperuricemia, reduced cardiovascular risk, especially in children. Further studies are needed to determine whether the improvements of reduced GH secretion by exogenous GH or GH releasing factors will have beneficial effects on hyperuricemia and other cardiometabolic risk in obesity children and adolescents.

## Methods

### Subjects

This study is a cross-sectional study performed between November 2013 and July 2017 at the Department of Pediatrics of The Second Hospital of Shandong University and Shandong Provincial Hospital affiliated to Shandong University. A total of 78 obese (69 boys and 9 girls) children and adolescents were enrolled in the current study. The inclusion criteria were: 1) obesity was defined by an individual BMI > 95 percentile for normal weight individuals of the same age and sex^30^; 2) the selective ages of obese subjects are all between 10 and 16; Participants were excluded from this study based on the following criteria: 1) children with organic hypothalamic disease, pituitary disease including dysfunction of the adrenal, thyroid, or gonadal axis; 2) type 1 or 2 diabetes mellitus, chromosome abnormalities or all sorts of syndromes; 3) children with renal disease, liver disease and other severe chronic illness; 4) children receiving medications that may affect endogenous GH secretion, lipid metabolism, blood pressure, insulin action, glucose and weight were ineligible for participation; 5) children with height less than the 3 percentile were also excluded. The control group consisted of 30 normal-weight (22 boys and 8 girls) healthy subjects. The obesity group and the control group were matched by age, gender and tanner stage.

The study was approved by the Ethics Committee of the Second Hospital of Shandong University and Shandong Provincial Hospital affiliated to Shandong University, and the written informed consent was obtained from all subjects’ parents. All methods were performed in accordance with approved guidelines and regulations.

### Clinical examinations

Height was measured to the nearest 0.1 cm on a standard height stadiometer and the weight was determined to the nearest 0.1 kg on a standard electronic scale. BMI was calculated as weight divided by height squared ((kg/m^2^). Height and BMI SDS were calculated according to reference values in Chinese children^30,31^. Pubertal developmental stage was determined according to Tanner criteria^32^. SBP and DBP were measured twice at the right arm after a 5 minutes’ rest in the supine position, and the medians of these two values were used.

### Laboratory measurements

Fasting blood samples were taken from all subjects after an overnight fast. Fasting blood was drawn for endocrine, metabolic markers. GH secretion was evaluated with two stimulation tests using arginine test (0.5 g/kg, with a maximum of 30 g) and levodopa test (10 mg/kg, with a maximum of 0.5 g). Serum GH levels were determined at baseline (0 minutes) and then at times 30, 60, 90, 120, 150 minutes after two stimulation tests. GH peak <10 μg/L was considered as relative GH deficiency. Serum GH levels were measured by chemiluminescence assay (Cobas E170, Roche Diagnostics, Germany). The intra- and inter-assay coefficients of variation (CVs) for GH were <5.0% and <6.0%, respectively. IGF-1 was measured by chemiluminescence assay (IMMULITE 2000, Siemens Health care Diagnostics, USA). Uric acid, TC, HDL-C, LDL-C, TG, fasting glucose and ALT, urea nitrogen and creatinine were detected by using Auto Biochemical Analyzer (AU5400, Beckman Coulter, Japan). Fasting insulin was determined by using a chemiluminescent immunometric assay (CobasE170, Roche Diagnostics, Germany). The intra- and inter-assay CVs were 4–8%. An oral glucose tolerance test (OGTT) (1.75 g/kg, with a maximum of 75 g) was performed in those who had fasting plasma glucose ≥ 5.6 mmol/L subjects. Thyroid function, adrenal function and gonadal axis function were performed on all participants to exclude hypothalamic-pituitary disease.

The criteria of International Diabetes Federation were used to define the metabolic syndrome^33^. Subjects in according with at least three of the following items were identified as metabolic syndrome: 1) obesity; 2) HDL-C < 1.03 mmol/L; 3)TG ≥ 1.7 mmol/L; 4) SBP ≥ 130 mmHg or DBP ≥ 85 mmHg; 5) fasting glucose ≥ 5.6 mmol/L. Hyperuricemia was defined with uric acid values > 5.5 mg/dL^34^. Insulin resistance was determined using HOMA-IR, which was calculated as fasting insulin × fasting glucose/22.5^35^.

### Hypothalamic pituitary MRI

Hypothalamic pituitary MRI was performed on a 3.0 T scanner (Siemens, Erlangen, Germany), with slice thickness of 3 mm. All obese children were scanned of sagittal and coronal planes with T1- and T2-weighted imaging.

### Statistical analysis

Variables that were normally distributed were expressed as mean ± SD and were compared using the Student’s t test. Peak GH on stimulation test, IGF-1, uric acid, TC, HDL-C, LDL-C, TG, HOMA-IR, insulin, ALT, urea nitrogen and creatinine were log transformed for statistical analysis, but values represent a back transformation to the original, and they were expressed as mean ± SD and compared using the Student’s t test. Categorical variables were compared by chi square test. Correlation between peak stimulated GH, clinical and metabolic variables were calculated by Spearman’s rank correlation coefficient analysis. Multivariate logistic regression analysis was performed to identify the association between peak stimulated GH and hyperuricemia, after controlling factors like age, gender, tanner stage and metabolic variables. Logistic regression analysis was also applied to demonstrate the association between peak stimulated GH and the metabolic syndrome after adjustment for age, gender and tanner stage. In order to avoid collinearity, we used HOMA-IR to represent fasting glucose and insulin. The results of logistic regression analysis were displayed as odds ratios (OR) with 95% confidence interval (CI). Differences of P < 0.05 were considered statistically significant. Statistical analyses were performed by SPSS version 20.0 (SPSS Inc. Chicago, USA).

### Data availability

The datasets used and/or analysed during the current study are available from the corresponding author on reasonable request.
